# Drivers of woody plant encroachment over Africa

**DOI:** 10.1038/s41467-018-04616-8

**Published:** 2018-06-11

**Authors:** Z. S. Venter, M. D. Cramer, H.-J. Hawkins

**Affiliations:** 1 Department of Biological Sciences, University of Cape Town, Private Bag X3, Rondebosch, 7701 South Africa; 2 Conservation South Africa, 301 Heritage House, Claremont, 7375 South Africa

## Abstract

While global deforestation induced by human land use has been quantified, the drivers and extent of simultaneous woody plant encroachment (WPE) into open areas are only regionally known. WPE has important consequences for ecosystem functioning, global carbon balances and human economies. Here we report, using high-resolution satellite imagery, that woody vegetation cover over sub-Saharan Africa increased by 8% over the past three decades and that a diversity of drivers, other than CO_2_, were able to explain 78% of the spatial variation in this trend. A decline in burned area along with warmer, wetter climates drove WPE, although this has been mitigated in areas with high population growth rates, and high and low extremes of herbivory, specifically browsers. These results confirm global greening trends, thereby bringing into question widely held theories about declining terrestrial carbon balances and desert expansion. Importantly, while global drivers such as climate and CO_2_ may enhance the risk of WPE, managing fire and herbivory at the local scale provides tools to mitigate continental WPE.

## Abstract (web summary)

Woody plant encroachment has important implications for habitat conservation and global carbon budgets, but its drivers require quantification. Here, Venter et al. report that encroachment is predominantly driven by human activities, changing weather conditions, fire, and herbivory.

## Introduction

Continental-scale changes in woody plant cover have been mapped for forests >5 m in height^1^, indicating an overwhelming deforestation trend induced by human land use^2^. A less well-known, yet equally important global trend is gradual woody plant encroachment (WPE), occurring in non-forest biomes^3^. In Africa, WPE has been identified as a concern for rangeland management since the early 20th century, and has the potential to reduce rangeland carrying capacities of wild and domestic grazers through the displacement of herbaceous forage by trees and shrubs. On the other hand, WPE may significantly contribute to forage for wild and domestic browsers, household fuel-wood provision, and may lead to increased carbon sequestration, with consequences for global carbon budgets and climate change^4^. In order to manage the effects of WPE on these diverse local and global ecosystem services, we need to understand what is driving it.

The drivers of WPE are poorly understood compared to those of deforestation where human-induced clearing is dominant. Rising atmospheric CO_2_^5,6^ and associated climatic changes, coupled with changing fire and herbivore management regimes, have been proposed as dominant drivers^3,4,7,8^. While homogenous global CO_2_ enrichment may enhance tree growth^9^, the trends in WPE are spatially variable, suggesting other local- or regional-scale drivers. For example, increases in rainfall have been shown to correlate with WPE, while the influences of trends in temperature are less clear^10^. Agriculturally induced transformation of Africa’s unique set of functional herbivore guilds^11^, and the alteration of fire regimes^12^ may shift systems into tree-dominated states at the local scale. However, quantifying these drivers at continental scales has been limited by the paucity of local-scale studies^3^ or continental analyses relying on low-resolution remotely sensed data^10^. The lack of spatially explicit measures of the magnitude and scale of WPE has made it difficult to draw generalised conclusions about its causes, and to identify the potential for the use of local drivers (i.e. fire, herbivory and human disturbance) as management tools to mitigate the putative effects of global (i.e. climatic) drivers on WPE.

We mapped change in woody plant cover excluding closed forest (more than 40% cover by trees taller than 5 m) at 30 m resolution for Africa over the past three decades. We considered a suite of potential drivers to explain this change, including CO_2_ as a global driver and other local- or regional-scale drivers that have received less attention (Supplementary Fig. 1). We report that non-forest biomes in Africa have undergone a net 8% increase in woody plant cover over the past three decades, although the magnitude and direction of this trend was spatially variable. During the same period there have been significant increases in CO_2_, rainfall and herbivory, and reductions in burned area. We develop a machine learning model to elucidate these complex correlations and find that a diversity of drivers other than atmospheric CO_2_ are able to explain 78% of the spatial variation in African woody cover change. WPE has been exacerbated by warming and wetting climates associated with global climate change, but local changes in fire, herbivory and direct anthropogenic disturbance (e.g. deforestation) predominate. Altering fire and herbivory management regimes thus has the potential to mitigate WPE.

## Results and discussion

### Broad-scale trends in woody plant cover

**Fig. 1:**
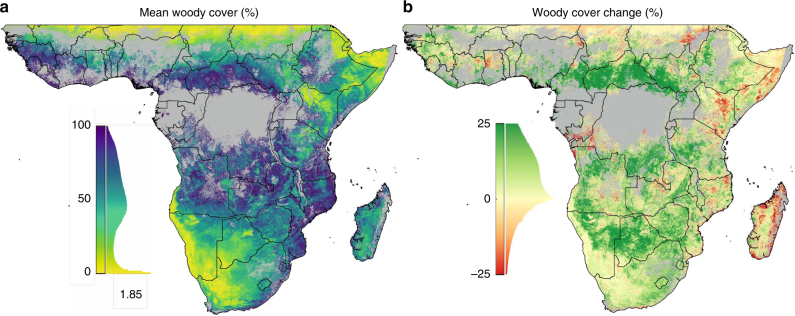
Woody plant cover dynamics over sub-Saharan Africa. Satellite observations of 30 years of fractional woody plant cover (**a**) reveal a dominant increasing trend (derived from the slope of the linear trend line between 1986 and 2016) (**b**). Histograms alongside colour scales indicate data distributions. Grey areas were masked from the analysis and represent urban surfaces, wetland, cropland, and forest (areas >40% cover by trees >5 m). Maps were constructed in Google Earth Engine^46^

**Fig. 2:**
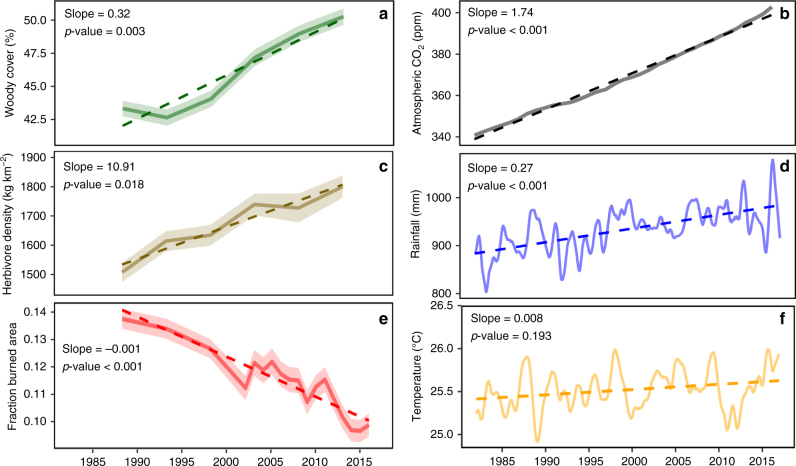
Time-series data for woody cover and select environmental covariates averaged over Africa. Solid lines represent the mean values and linear trend lines are indicated with dashes. Using 0.5° grid cells as replicates (*n* = 6255), 95% confidence interval ribbons have been included in **a**, **c** and **e**. The slope of the trend line and *p*-value of the linear regression are displayed for each plot. Herbivore density and burned area have been hind- and forecast using methods outlined in the supplement. Solid lines for rainfall (**d**) and temperature (**f**) indicate inter-annual trends once seasonality has been removed, whereas this is not the case for CO2 (**b**). Inflection points for lines in **a** and **c** are plotted at the median timepoint for each epoch

Over the past three decades, 7.5 million km^2^ (55%) of non-forest biomes (see data mask in Supplementary Fig. 2) in sub-Saharan Africa underwent significant net gains in woody plant cover (Figs. 1, 2a, and Supplementary Fig. 3). This is more than triple the 2.2 million km^2^ (16%) significant decrease in woody plant cover, confirming local-scale studies indicating increases in WPE over the last century^3^. Woody cover loss was prevalent in parts of the Sahel, East Africa and much of Madagascar, but WPE dominated the central-interior of Africa. Countries exhibiting a mean fractional increase >30% were Cameroon, Central African Republic, South Sudan, and Uganda (Supplementary Table 1). Almost all other counties experienced net encroachment, with only Congo, Kenya, Madagascar, Niger and Somalia undergoing a net decline in woody cover. The highest rates of encroachment occurred in areas with moderate initial woody cover (i.e. 30–60%) in 1986 (Supplementary Fig. 4). Areas with more than 75% initial cover experienced highest rates of loss, probably due to human-induced clearing (e.g. Supplementary Fig. 3). There was little difference between WPE inside (13.9%) and outside of (12.5%) protected areas. Encroachment trends were lowest in shrublands (3.5 ± 0.4% increase) and highest in Caesalpinioid savannas (20 ± 0.4% increase), but were pronounced across all vegetation types (Supplementary Fig. 5), indicating that the drivers of this change are globally available, but act regionally allowing WPE in some areas and deforestation in others.

### Drivers of woody plant cover change

The widespread trend in WPE correlates with a significant rise in atmospheric CO_2_ and rainfall (Fig. 2b, d), but also a significant increase in herbivore densities and decline in burned area (Fig. 2c, e). To avoid drawing conclusions about drivers of WPE from such continental-scale correlations (Fig. 2) without acknowledging the spatial variation in trends (i.e. some areas have increased in rainfall or woody cover while others have decreased), we employed the established machine learning technique of boosted regression tree (BRT) modelling^13,14^ to investigate the relative importance of and interactions between a set of >60 explanatory variables (climatic, edaphic and disturbance) and woody cover change.

**Fig. 3:**
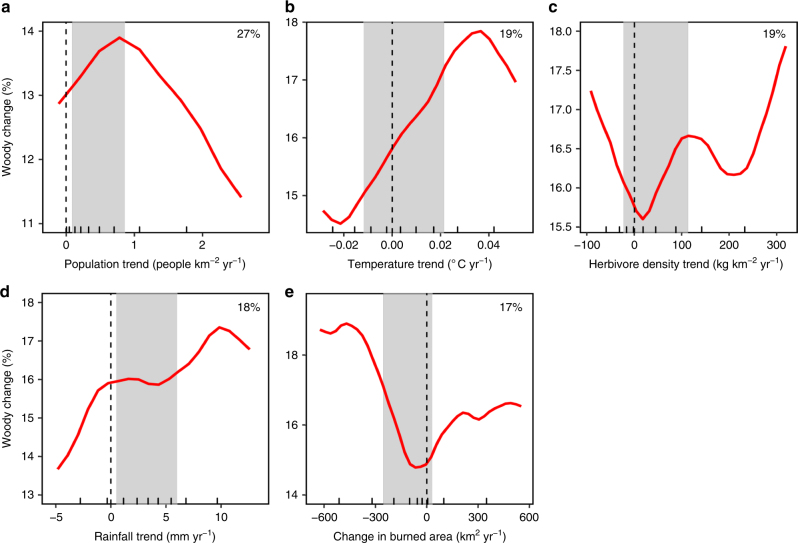
Most important drivers of woody plant cover change. **a**-**e** Boosted regression tree partial dependence of fractional woody cover change on selected explanatory variables, when accounting for the average effect of all other driver variables (see Supplementary Fig. 7). The red lines are smoothed representations of the responses, with fitted values (model predictions based on the original data) for each 0.5° grid cell over sub-Saharan Africa. The trend of the line, rather than the actual values, describes the nature of the dependence between response and explanatory variables. Small bats on the x-axis represent data deciles, and grey bands indicate data between the 25th and 75th percentile. The x-axis was clipped to the 5th and 95th percentile to highlight trends in the bulk of the data. The full model explained 51% of the total deviance in woody cover change, and the relative contribution (%) of each explanatory variable is indicated

Our final model explained 78% of the deviance in spatially explicit woody cover trends. WPE expresses a hump-shaped response to human population growth (Fig. 3a). At high population growth rates, WPE was inhibited, presumably due to clearing, emphasising that deforestation trends^1^ are not limited to the forest biome. Low population growth rates had a negligible effect on curbing WPE, potentially due to a covariance with human-induced landscape fragmentation and the subsequent reduction in fire spread^12^. Local disturbances by fire and herbivory are known to maintain open savannas in areas that could climatically support closed-canopy forest^15^. Our analysis confirms that local disturbance patterns can have continental consequences for WPE and are of equal importance to edaphic and climatic variables in explaining the spatial variation in woody cover change (Supplementary Figs. 6 and 7). Large reductions in burned area in Africa, consistent with the global trend^16^, have driven larger WPE rates (Fig. 3e). Decreases in fire reduces tree mortality and consequently reduces competition from the grass layer and facilitates tree recruitment which further reduces the grass fuel load for fires, creating a negative feedback loop^17^. The bulk of the data for trends in herbivory suggest that increasing herbivore intensity exacerbates WPE (shaded area in Fig. 3c). Grazing herbivores, which dominate most African rangelands^18,19^, reduce grass competition with woody plants and reduce fuel loads for fires, thereby releasing woody plants from the fire trap^8,20^. However, WPE might also be facilitated in areas with large declines in herbivory (Fig. 3c). These contradictory herbivore-induced effects on WPE are likely due to differing livestock management contexts coupled with the widespread loss of mid-Holocene herbivore functional guilds, such as browsers^18,21^. Browsers play an important role in regulating woody plant populations through direct mortality (e.g. elephant impact^11,22–24^) or by inhibiting shrub and tree growth rates and thereby increasing vulnerability to fire^17,25^. Indeed, we found that areas with high browser densities experienced lower encroachment rates (Supplementary Fig. 8a). In contrast, grazers reduce fuel loads for fire and thus enhance WPE^17^; however, we found that extreme grazer densities may inhibit WPE (Supplementary Fig. 8b). One possible way that high grazer densities may reduce WPE is through consumption and trampling of coppicing and young woody plants.

Areas experiencing increases in rainfall underwent greater WPE than those where rainfall has decreased (Fig. 3d), confirming rainfall as a potent determinant of tree cover^26,27^. Although rises in temperature have been shown to enhance WPE at local scales through declines in frost-induced tree mortality, the regional-scale interaction between changes in temperature and woody cover are less well understood for Africa^8^. Here we show that changes in WPE with rising temperatures mirrored the effect of increases in rainfall (Fig. 3b), suggesting that WPE may be set to continue under global warming scenarios. The detrimental effects of increased transpiration and drought stress under warmer temperatures may be mitigated by wetter climates and enhanced water use efficiency induced by rising atmospheric CO_2_^7,28^. Experimental evidence also exists for increased seedling establishment under warmer climates for some savanna woody species^29^.

Apart from the interactive effect with temperature and water use efficiency, rising atmospheric CO_2_ levels might contribute to continental WPE through enhanced C_3_ woody plant photosynthetic rates and post-fire resprouting capabilities, relative to C_4_ grasses^30,31^. The lack of spatial variability in atmospheric CO_2_ trends precluded it from being incorporated into our model. Notwithstanding, the changes we observe between 1986 and 2016 might reflect the legacy effects of post-industrial revolution CO_2_ trends, although the shape of continental trend lines suggests that the temporal variation in WPE rate is not directly linked to that of CO_2_ (Fig. 2a, b). While experimental studies have noted a positive growth response in trees to elevated CO_2_^9,32^, the strength of this response relative to herbaceous plants is variable, especially when considered in isolation from nutrient limitations and competitive interactions present in natural systems but commonly absent in experimental set-ups^33,34^. While CO_2_ may contribute to WPE, the global trend in atmospheric CO_2_ has not led to homogenous trends in WPE (Fig. 1b). Thus the other climatic and disturbance drivers assessed here are important in determining the direction of vegetation change and determining the magnitude of WPE.

### Implications

The widespread continental increase in woody plants shown here corroborates global trends of increasing leaf area index^35^ and vegetation greenness^36^ in semi-arid areas, thereby challenging the long-held desertification narrative^37^. The inclusion of spatially explicit greening trends into global carbon budgets have previously relied on low resolution (>250 m) estimates of net primary productivity in semi-arid areas^38^. The present dataset of decadal woody cover change might aid in more accurately quantifying the extent to which WPE contributes to the global carbon sink, potentially offsetting the carbon losses from deforestation. Despite the potential benefits to the global carbon budget, the local-scale disadvantages (e.g. reduced grazing capacity) and their effects on rural livelihoods has motivated substantial governmental investment into clearing alien and native invasive woody plants (e.g. ca.100 million US$ per annum in South Africa)^39^. Initial indications from our models suggest that WPE management interventions will be most needed in areas that are expected to increase in temperature and rainfall under future climate change scenarios. More importantly, manipulating local disturbance patterns has the potential to override climatic effects and significantly mitigate WPE. Management interventions may include increasing fire using heterogenous management regimes^40^, or through rewilding savannas with historical herbivory pressures^11,21^, and diversifying herbivore functional guilds by incorporating more browsers^18^. Thus, while global drivers such as climate and CO_2_ may enhance the risk of WPE, the realisation of WPE is largely dependent on management decisions.

## Methods

### Fractional woody cover prediction

The study area included sub-Saharan Africa, totalling 20.5 M km^2^, equivalent to 22.8 billion Landsat pixels. Woody plant cover was defined as fractional woody cover of 30 × 30 m squares, defined by the Landsat pixel grid. The dynamics of tree cover change have been comprehensively explored using remote sensing techniques for forest biomes^1^. Given that the potential for WPE to occur in areas already saturated with tree cover is negligible and that our aim was to investigate WPE and its drivers, we excluded the forest biome from our analysis. Pixels with >40% cover by trees of >5 m in height were considered as closed forest^41,42^ and excluded using data from the Global Land Cover Facility^43^. Tree cover may be unable to fully distinguish forests from densely wooded savannas^44^; however, these ecotonal boundary areas are relatively small compared to the total area occupied by true non-forest biomes. Thus, the erroneous masking of densely wooded savannas was expected to have little effect on the continent-wide analysis. Forestry areas were defined as pixels that have both lost and gained woody cover between 2000 and 2015 using global forest cover change data derived from Hansen et al. 2013^1^ and were excluded from our analysis. Urban surfaces, water, wetland, cropland, and natural-cropland mosaics were also excluded from the analysis using the MODIS landcover product^45^. This combined pixel mask (Supplementary Fig. 2) was applied to all Landsat- and MODIS-derived data in this analysis.

The remote sensing analysis was performed using the Google Earth Engine cloud computing platform for earth observation and data analysis^46^. The near-complete set of Landsat surface reflectance data available for Africa (1986–2016) from the USGS Earth Resources Observation and Science archive^47^ were analysed to identify change in fractional woody cover. We analysed 6 epochs of Landsat data between 1986 and 2016 (Supplementary Fig. 1). Landsat 5 Thematic Mapper (TM) was used for the 1986–1991, 1991–1996 and 1996–2001 epochs. Landsat 7 Enhanced Thematic Mapper Plus (ETM+) was used for the 2001–2006, 2006–2011 and 2011–2016 epochs. Data gaps in the 2011–2016 epoch were filled by merging the Landsat 7 ETM+collection with the Landsat 8 Operational Land Imager (OLI) collection using published cross-calibration coefficients for surface reflectance^48^. A cloud mask and confidence quality assessment data were used to create cloud-free image collections, which were used to derive per-pixel time-series spectral metrics for each epoch. Temporal reflectance data were derived from visible, near infrared, and shortwave infrared bands, as well as three vegetation indices, namely normalised difference vegetation index^49^, soil-adjusted vegetation index^50^, and enhanced vegetation index^51^. Vegetation indices have been used extensively in vegetation cover mapping and landcover classification^52^. Time-series metrics derived from these included the minimum, maximum and selected percentile values (10, 25, 50, 75 and 90% percentiles) and the mean reflectance values for observations between selected percentiles (10–25%, 25–50%, 50–75%, 75–90% and 25–75%). Similar time-series metrics have been successfully used in forest cover mapping using Landsat data^1,53,54^. To further assist in differentiating between woody and herbaceous cover, which have different phenological metrics^55^, we derived the variance and range in vegetation indices over time for each epoch.

Time-series metric data were used to train a Random Forest (RF) regression model to predict fractional woody plant cover for each 5-year epoch (Supplementary Fig. 1). RF is a supervised classification and prediction tool has that has been extensively used because it avoids overfitting and can incorporate non-parametric data^56^. Training data were derived from image interpretation methods using very high spatial resolution images derived from Google Earth. We generated 4000 randomly scattered 30 × 30 m sampling quadrats, aligning with the Landsat pixel grid, within the unmasked areas for the given Landsat epoch collection. We manually classified the fractional woody plant cover of each sampling quadrat by identifying woody plant canopies using texture, colour and canopy shadows as identification cues (Supplementary Fig. 9). We estimated the woody plant cover to the closest percentile class (0, 0.25, 0.5, 0.75, 1). Sampling quadrats were excluded if the image acquisition date fell outside of the epoch date range or if there was any uncertainty in designating a fractional woody plant cover value. A separate RF classifier was trained for Landsat 5 TM, Landsat 7 ETM+, and gap-filled Landsat 7 ETM+ with Landsat 8 OLI collections. RF accuracy assessment traditionally employs internal cross-validation between in-bag samples used to train the trees, and out-of-bag samples used for model validation^57^. However, recent literature suggests internal cross-validation may over-estimate model accuracy, and suggest validation against an testing dataset independent from that used in model construction^56,58^. Our RF regression models produced high accuracies when using both internal and independent hold-out datasets for validation (Supplementary Table 2).

The RF models were used to predict fractional woody plant cover across Africa at 30 m resolution for each epoch. Pixel-level change was defined by the slope of the linear regression between fractional woody cover and year. This is the same metric of change employed by other remote sensing analyses of forest cover change^1^. Although the response variable in the linear regression was bounded (i.e. proportional woody cover), the model assumptions were checked and satisfied, thus data were not transformed prior to fitting the model. Nevertheless, the analysis of drivers of woody cover change was performed on both untransformed and logit-transformed woody cover data, and both yielded similar results. Estimates of data quality were calculated for each pixel based on the number of available Landsat timepoints for the linear regression, and the total number of pixels used to derive time-series metrics (Supplementary Fig. 10).

### Environmental covariates

To explain the change in fractional woody cover we obtained a broad set of climatic, edaphic, biotic, and demographic explanatory variables (Supplementary Figs. 6 and 7). All variables were sourced and analysed within the Google Earth Engine platform, except for herbivore density, protected area status and soils data, which were obtained from sources documented below and analysed within R^59^ and QGIS^60^.

High temporal resolution climatic data were obtained from the Global Land Data Assimilation System (GLDAS) produced by NASA at 0.25° every 3 h between 1986 and 2016^61^. Variables included were surface temperature, air temperature, rainfall, potential evaporation rate, soil moisture and wind speed. Additional rainfall data were obtained from the Tropical Rainfall Measuring Mission^62^ and Climate Hazards Group (CHIRPS)^63^ for comparison with GLDAS. Annual counts of extreme rainfall events, defined as any 5-day rainfall amount that exceeded the 95th percentile of all measurements for that gird cell^64^, were calculated. Rainfall variability was calculated as the standard deviation across both yearly and 5-hourly time series. The extent to which rainfall is evenly distributed though the year was calculated as the precipitation concentration index^65^ using data from the CHIRPS dataset. For each variable, we calculated the long-term average and the slope of the linear trend over time. WorldClim rainfall and temperature min, max, mean values for the driest, wettest, warmest and coldest quarters, and seasonality were also included^66^.

Mid-troposphere daily CO_2_ concentration data at 2 × 2.5° resolution were obtained from Atmospheric Infrared Sounder between 2010 and 2017^67^. The means and trends were calculated per grid cell, but after consideration were not included in the modelling procedure for the following reasons: the data were collected at lower spatial resolution than all other explanatory variables; they were collected for the mid-troposphere and thus the relevance to ambient ground-level CO_2_ was questionable; and, unlike other bio-climatic variables, the range in the means (2 ppm) and temporal trends in CO_2_ (0.35 ppm yr^−1^) concentrations were very small (Supplementary Fig. 11) in comparison to the CO_2_ enrichment values necessary (>160 ppm) to induce significant changes in woody plant growth^34^. An attempt was also made to include the long-term CO_2_ trend in the model, however, because this is spatially homogenous it had very low explanatory power and was thus excluded.

Edaphic data were derived from the ‘SoilsGrid 1 km’ global dataset^68^. These included depth to bedrock (R horizon); bulk density (kg m^3^); cation exchange capacity (cmol kg^−1^); clay and sand content (% gravimetric); soil organic carbon content (g kg^−1^) and pH (in H_2_O). The data for six soil depths were aggregated by depth-weighted averaging (i.e. averaged by weighting values for each depth-interval). Digital elevation at 30 m resolution from the Shuttle Radar Topography Mission^69^ was used to calculate a terrain ruggedness index^70^, which measures the sum change in elevation between a pixel and its eight neighbouring pixels.

Herbivory data were supplied by Archibald and Hempson^71^ at quarter degree resolution. These included modelled grazer, browser, mixed feeder and total herbivore densities using the FOA livestock data^19^ and indigenous wildlife census data from reserves across Africa. To obtain a change layer for herbivore density, we constructed a BRT model (see methods in following section) to hind- and fore-cast herbivore densities. The FAO reference year used in the dataset was 2005, thus the 2001–2006 epoch was used as the starting point for hind- and fore-casting. The model was able to explain 72% of the total deviance in herbivore density. Explanatory variables included population density, normalised difference vegetation index, longitude, latitude, temperature, and rainfall which contributed 25, 24, 22, 21, 5, and 3% to the explanatory power of the model, respectively. The slope of the linear trend in modelled herbivore density was calculated for each 0.5° square. Despite the uncertainty in deriving herbivory trends, we found that its removal/addition in woody cover change models did not unduly influence model explanatory power. Removing change in herbivory from the model presented in Fig. 3 reduced the explanatory power by only 4%.

Fire data from the MODIS (MCD45A1.051) burned area monthly product at 500 m resolution^72^ were used to derive the annual average and annual trend in mean annual burned area, fire frequency, and burn date per 0.5 × 0.5° square between 2000 and 2017. Due to technical problems on the MODIS satellite experienced during 2001^73^, we decided to exclude burned area for 2001 in our analysis. To derive trends in fire data that are representative of the study period (1986–2016), we followed the same approach as with herbivory and hind-cast fire data using a BRT model. The model, trained on the mean fire data between 2000 and 2017, was able to explain 70% of total deviance. For further validation, a separate model, trained on 2006–2011 mean data, was used to predict burned area for 2001–2006 and 2011–2016 mean data. The adjusted *R*^2^ of the linear regression between observed and predicted burned area for 2001–2006 and 2011–2016 was 0.66 and 0.72, respectively, thus corroborating the predictive capability of the model used for hindcasting. Explanatory variables included in the model were latitude, normalised difference vegetation index, population density, longitude, rainfall, and temperature, which contributed 24, 22, 17, 15, 14, and 8% to the explanatory power of the model, respectively. The equivalent analyses were conducted on fire intensity data from the Fire Information for Resource Management System dataset^74^. All fires that fell within the data mask (Supplementary Fig. 1) used in the woody plant cover analysis were excluded.

We determined the proportion of each 0.5° square covered by protected areas using data from Protected Planet (www.wdpa.org)^75^. Quarter degree squares were classified into vegetation type^76^, ecoregion and biome^77^ based on the centroid of each grid cell. The average and trend in African population density between 2000 and 2015 at 1 km resolution was obtained from the Gridded Population of the World, Version 4 (GPWv4) dataset^78^.

### BRT modelling

To assess the interactions between explanatory variables and fractional woody cover change, we used BRTs (Supplementary Fig. 1) which have been used extensively in ecological studies to analyse complex systems, including drivers of woody plant cover^26^. BRTs are an advanced form of machine learning that iteratively fit and combine multiple regression tree models to improve predictive performance^13^. An advantage of BRTs is their ability to ingest explanatory variables of multiple classes to model complex interactions with a given response without making assumptions about variable interactions, as is often the case with other forms of linear and non-linear modelling^13^. All BRTs were fitted in R^59^, using the ‘dismo’ library following the procedure outlined by Elith et al.^13^.

Variables used in the modelling exercise were aggregated up to a common spatial resolution of 0.5°. Raw data with a resolution >500 m were resampled to 0.5° using bilinear resampling, and those with a resolution ≤500 m were reduced to the mean value per 0.5° grid cell. Data points were assigned a quality weighting based on the 30 m per-pixel quality layers (Supplementary Fig. 10) and the number of unmasked pixels per 0.5° cell. This was used as a weighting variable by assigning it to the “site.weights” call in the BRT model to prevent low quality data with small samples sizes from having an undue influence on the model fitting and prediction. Data with a quality score less than the 0.25 percentile value were excluded from the BRT analysis.

Combined and separate models were fitted with explanatory variables termed “drivers” and “facilitators” of woody cover change. We distinguished between explanatory variables with a temporal component (e.g. slope of linear trend in precipitation) and called these drivers, and those without a temporal component (e.g. average precipitation) and called these facilitators of WPE. Prior to fitting the models, we identified a limited set of strongly collinear variable groups with an *r*>0.7^79^ (Supplementary Fig. 12) and removed variables within these groups that were deemed less likely to be influential for woody cover change. Nevertheless, the excluded collinear variables were kept in mind during the analysis of model results. Further, no trend variables were collinear, making interpretation of the model with drivers of woody cover change (i.e. Fig. 3) simpler. Following parameter optimisation, we used family = Gaussian, tree complexity = 5, learning rate = 0.01, bag fraction = 0.5 and cross-fold validation = 10 as model parameters. The initial BRT models were simplified using procedures described by Elith et al.^13^, and only the variables with the highest explanatory power were included and analysed for interactions with change in fractional woody cover. To ensure that the BRT results were not a product of chance, we randomly assigned woody cover change values for all 0.5° grid cells and re-ran the model. The model failed to resolve, thus confirming the initial results were not a product of chance. The relative importance of predictors was determined based on the number of times it was selected for splitting, weighted by the squared improvements to the model, averaged over all trees^80^. Our final models for the simplified set of 31 combined, 25 facilitator (Supplementary Fig. 6) and 12 driver (Supplementary Fig. 7) explanatory variables explained 78%, 75% and 51% of the total deviance in woody cover change, respectively. Further to this, we reduced the trend model to include only drivers that are most often inferred in woody encroachment literature (i.e. fire, herbivory, population density, rainfall and temperature trends). This final model included five predictors and explained 34% of the total deviance in woody cover change.

### Data availability

Data that support the findings of this study are available from the corresponding author upon request. The woody cover change raster is available at full resolution online via Google Earth Engine upon request.

## Electronic supplementary material

Supplementary Information

Peer Review File
