# Supplementary material for: Drivers of woody plant encroachment over Africa
Source: Nat Commun. 2018 Jun 11;9:2272. doi: 10.1038/s41467-018-04616-8 (PMC5995890; doi:10.1038/s41467-018-04616-8)
Supplement: Supplementary file 1 — Supplementary Information [file 41467_2018_4616_MOESM1_ESM.pdf]

Drivers of Woody Plant Encroachment Over Africa

Supplementary information

Venter et al.

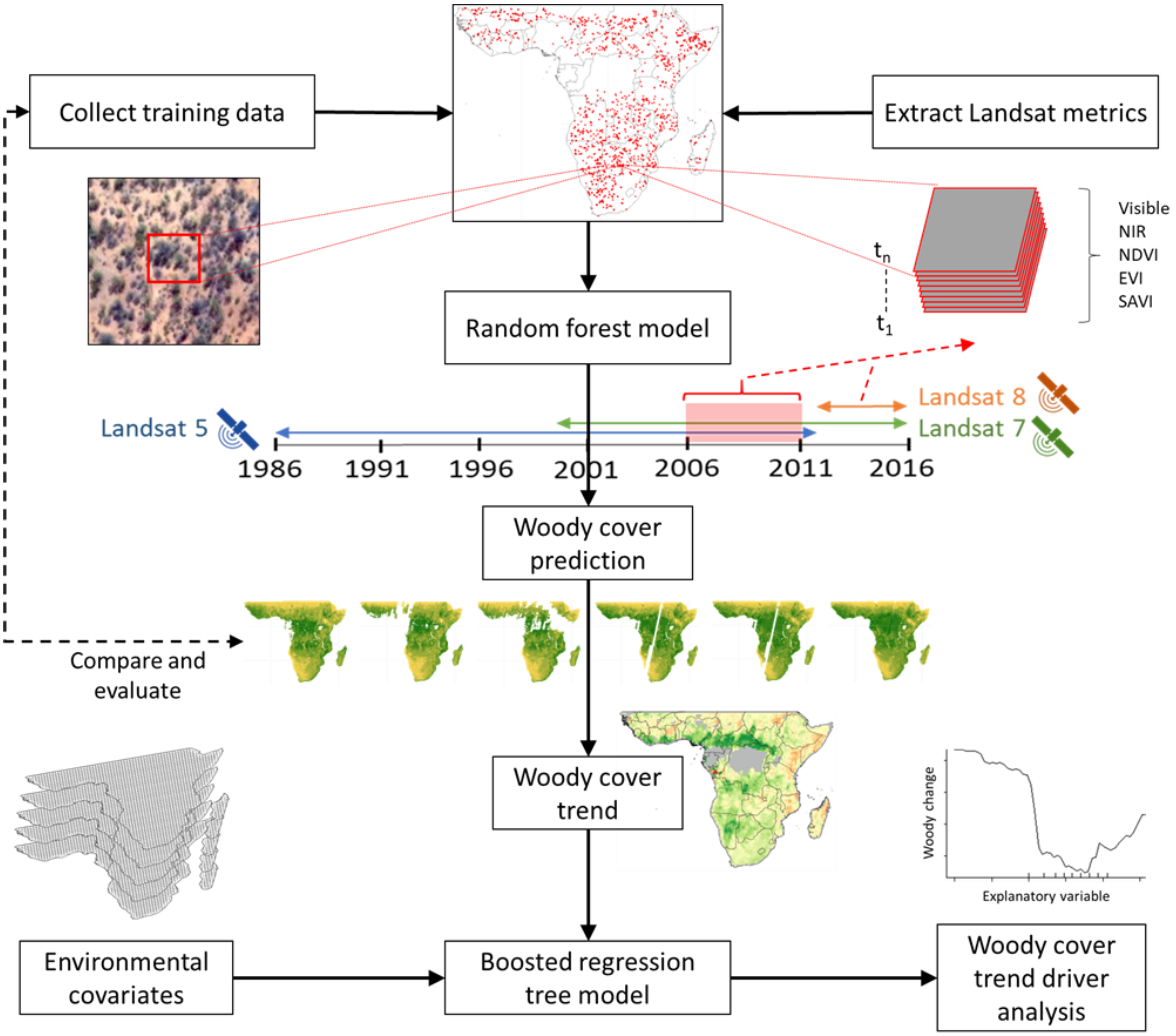

Supplementary Figure 1

Data processing flow for woody cover prediction and environmental covariate analysis. Aerial photograph: Google, DigitalGlobe. Maps constructed in Google Earth Engine [1].

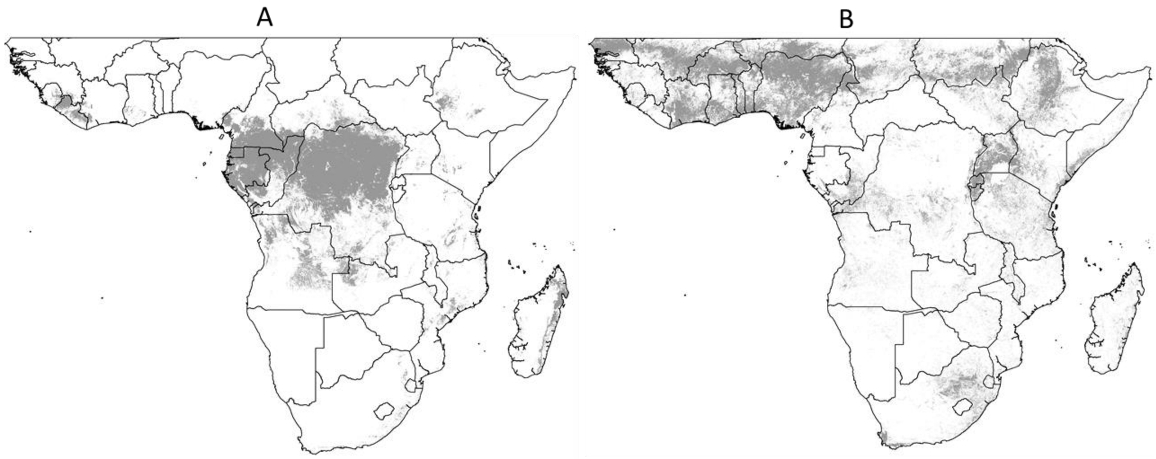

Supplementary Figure 2

Data masks, represented in grey, include Landsat-derived forest and forestry cover (A), MODIS-derived urban, water, wetland, cropland, and natural-cropland mosaics (B). Maps constructed in Google Earth Engine [1].

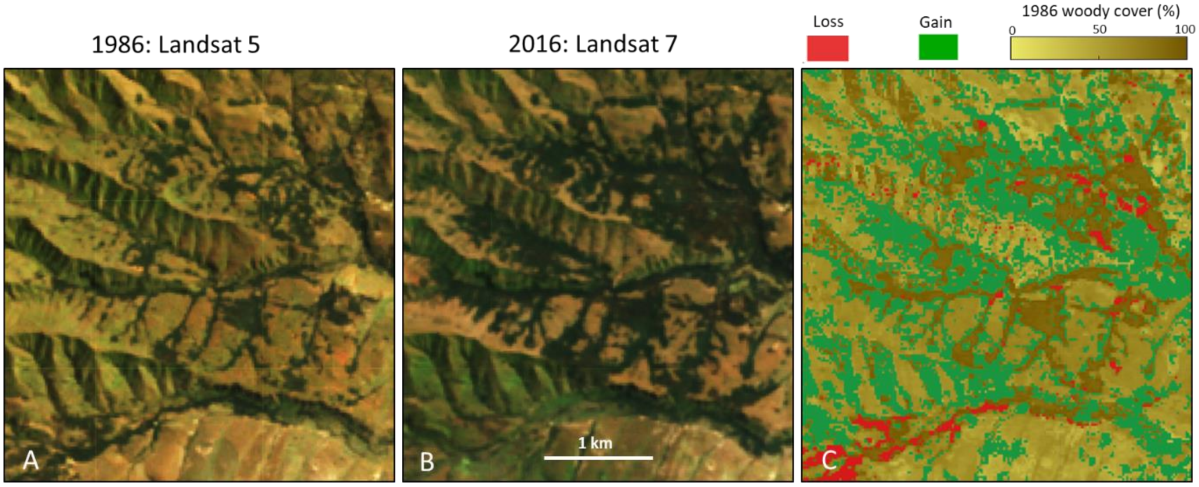

Supplementary Figure 3

Landscape-scale example of fractional woody plant cover change. Landsat true-colour composites of Mariazell Mission in the Eastern Cape of South Africa are shown for comparison over time (A, B). Gain and loss represent areas with > 50% change (C). This communal rangeland has been invaded by the exotic *Acacia mearnsii*, however, brush clearing efforts, initiated by the Working for Water Programme, and implemented through Conservation South Africa with the aim of rehabilitation of grazing capacity, are evident in red. Landsat-5 and -7 images courtesy of the U.S. Geological Survey.

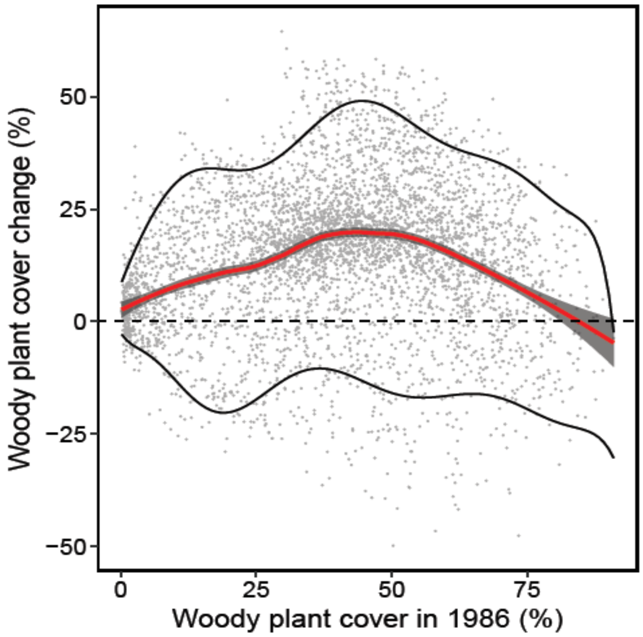

Supplementary Figure 4

Woody plant cover change relative to the initial fractional cover in 1986 for each 0.5° grid cell over sub-Saharan Africa. A loess regression line (red), its 95% confidence intervals (grey ribbon), and the 5th and 95th quantile regression lines (black) are indicated.

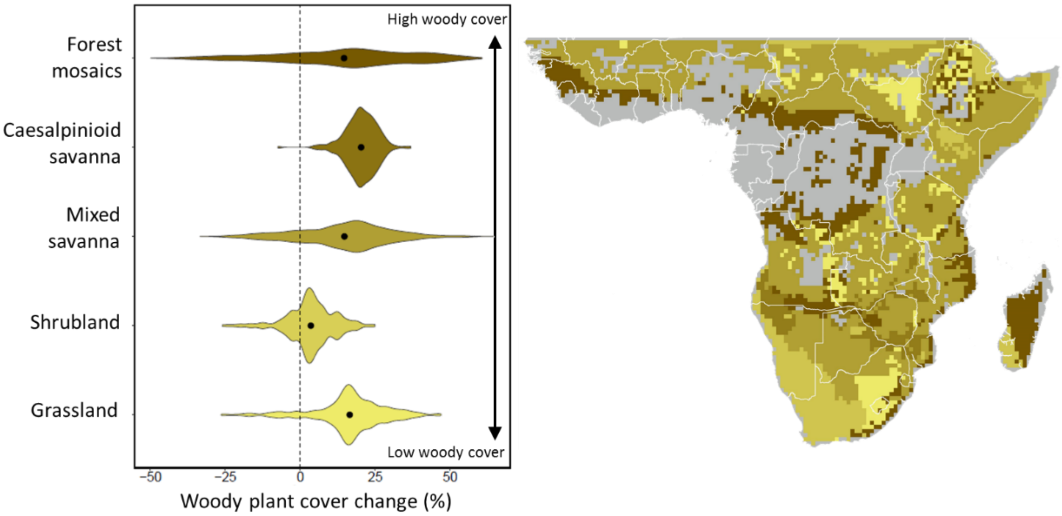

Supplementary Figure 5

Violin plot distributions of woody cover trends for different vegetation types (left). Vegetation types, plotted spatially for reference (right), are based on those defined by White [2], and are ordered and colored categorically by increasing fractional woody cover. Grey areas were masked from the analysis and represent urban, wetland, cropland, and forest (areas >40 % cover by trees >5 m). Maps constructed in Google Earth Engine [1].

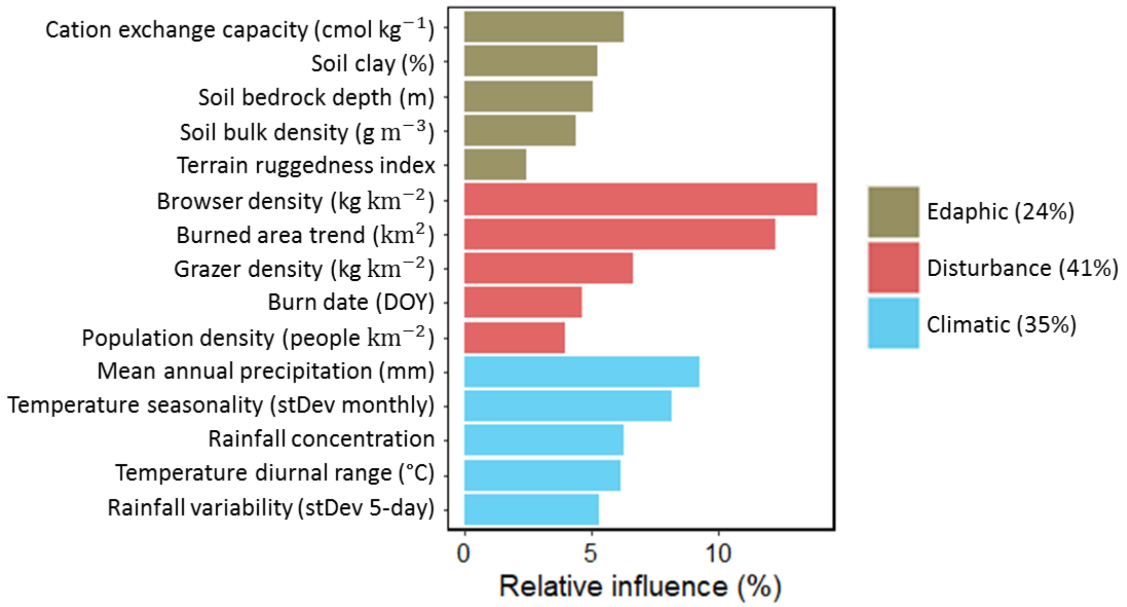

Supplementary Figure 6

The percentage contribution of the five most important facilitator variables (edaphic, disturbance, and climatic temporal means) as predictors employed in the final boosted regression tree model explaining the spatial variation in woody plant cover change. Summed contributions are indicated in the colour key. The model was able to explain 75% of the total deviance in woody cover change.

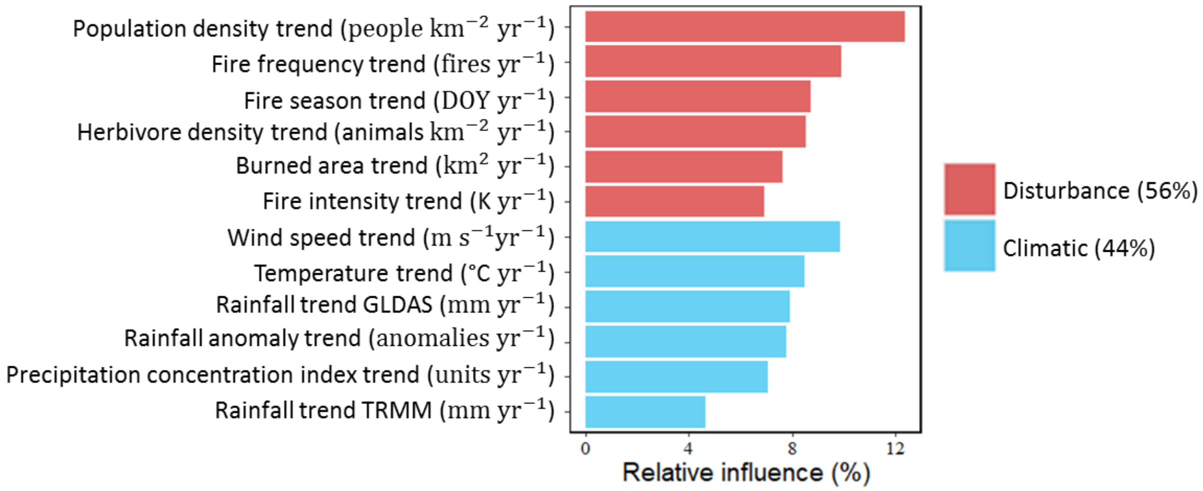

Supplementary Figure 7

The percentage contribution of the most important driver variables (climatic and disturbance trends) as predictors employed in the final boosted regression tree model explaining the spatial variation in woody plant cover change. Summed contributions are indicated in the colour key. The model was able to explain 51% of the total deviance in woody cover change.

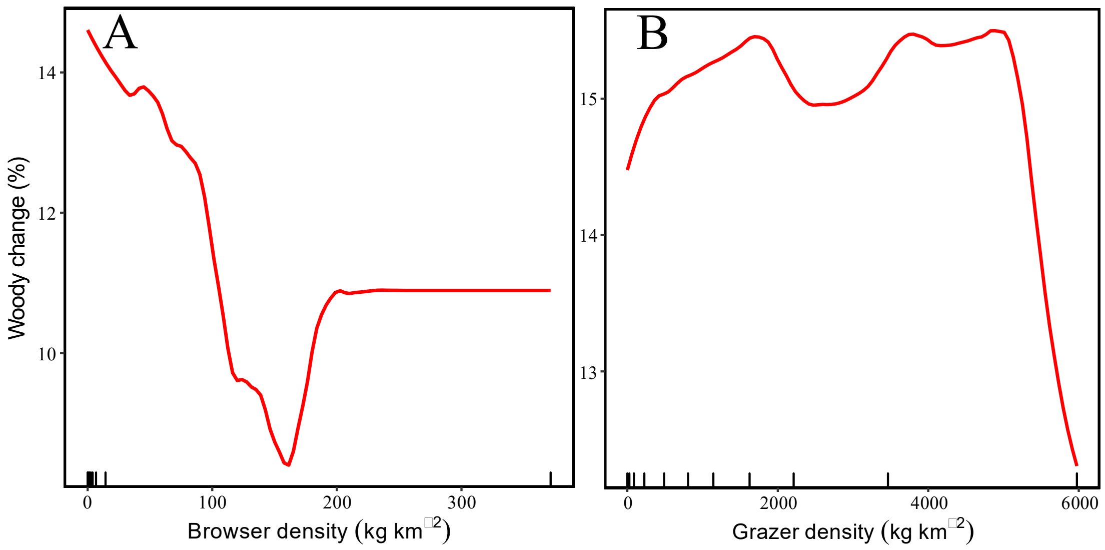

Supplementary Figure 8

Boosted regression tree partial dependence of fractional woody cover change on browser (A) and grazer (B) densities when accounting for the average effect of all explanatory variables. These contributed 8.6 and 5.6% to the final model combining drivers and facilitator variables which explained 78% of the deviance in woody cover change. The red line is the smoothed representation of the response, with fitted values (model predictions based on the original data) for each 0.5° grid cell over sub-Saharan Africa. The trend of the line, rather than the actual values, describes the nature of the dependence between response and explanatory variables. Small bats on the x-axis represent data deciles.

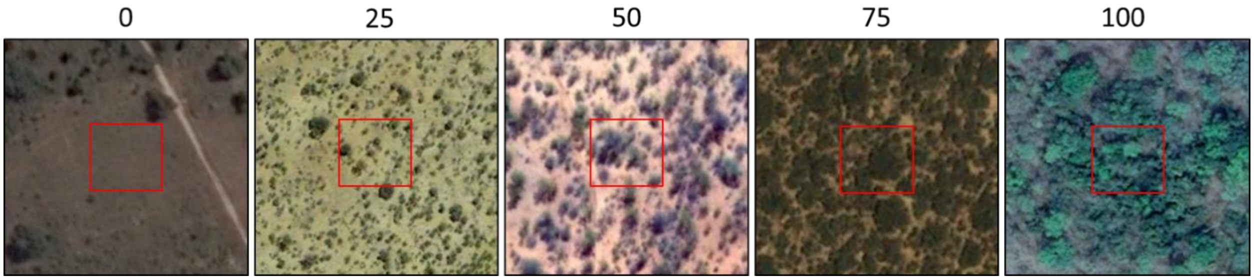

Supplementary Figure 9

Google Earth image examples for each fractional woody plant cover (%) category with 30 x 30 m sampling quadrat overlaid in red. Aerial photographs: Google, DigitalGlobe.

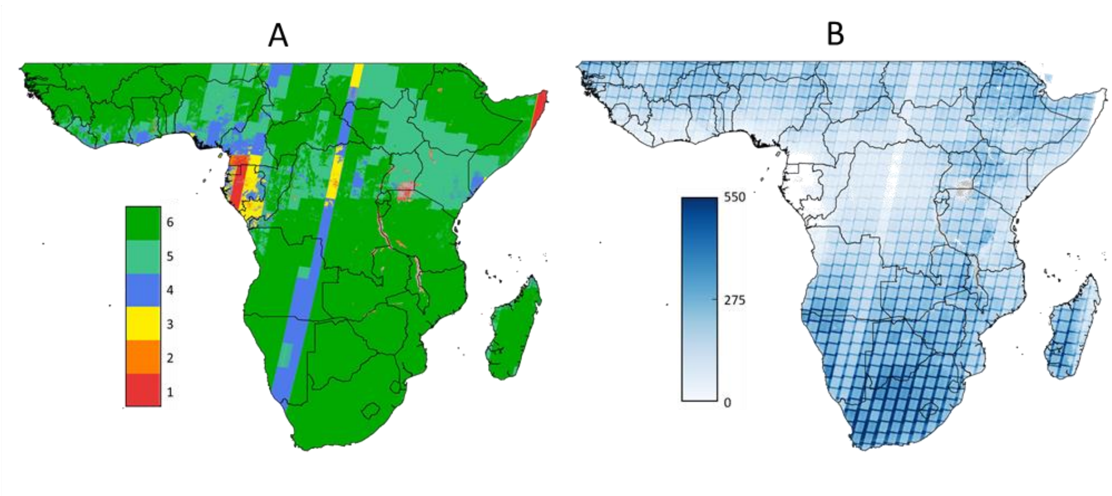

Supplementary Figure 10

Quality layers for fractional woody cover change prediction. Number of epochal time-points available for linear regression (A). Total number of cloud-free Landsat pixels between 1986 and 2016 (B). Maps constructed in Google Earth Engine [1].

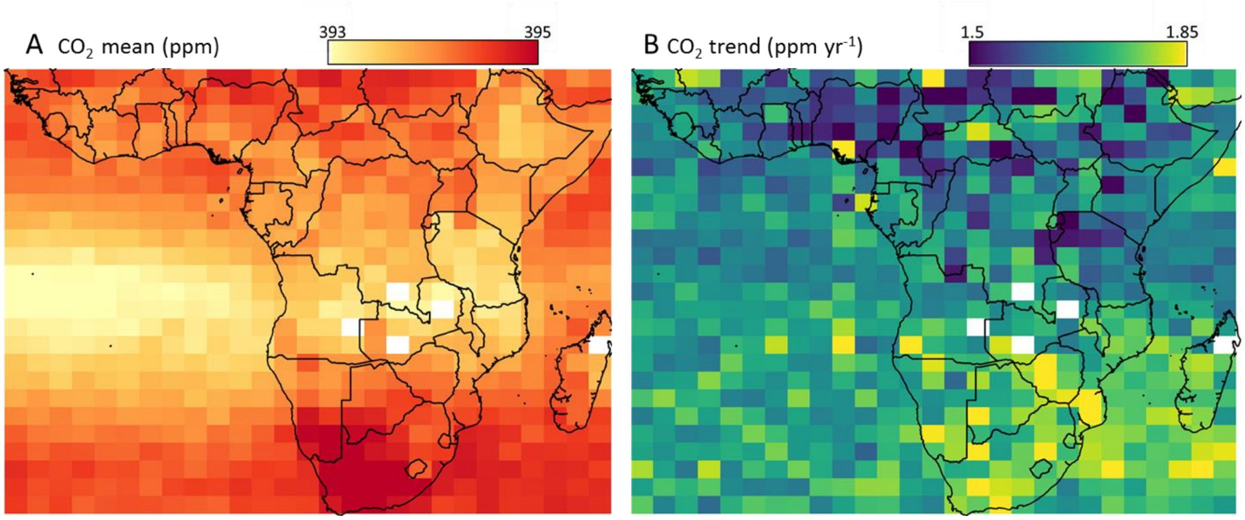

Supplementary Figure 11

Mean (A) and trend (B) in mid-troposphere atmospheric CO_2 concentrations (parts per million) for the period 2009 – 2017. The ranges of the spatial variation in CO_2 means and trends were 2 ppm and 0.35 ppm yr^{-1}, respectively. Maps constructed in Google Earth Engine [1].

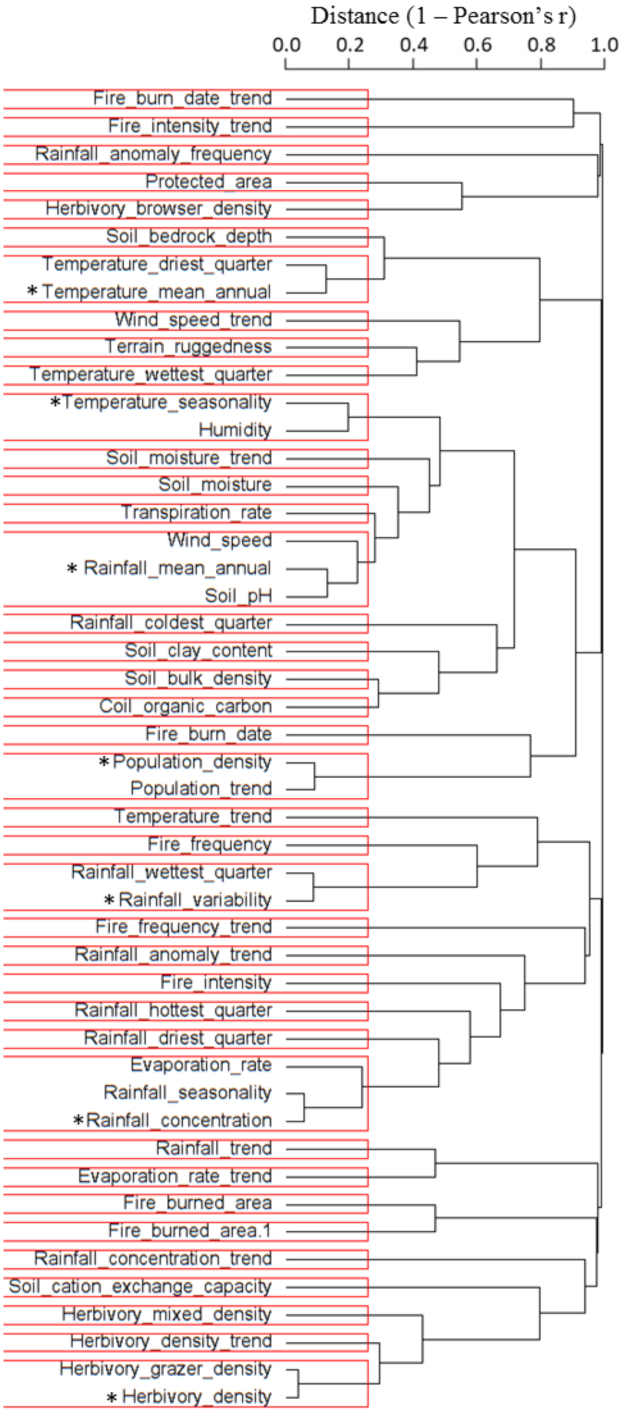

Supplementary Figure 12

Collinearity plot of explanatory variables used in the BRT model. Groups of variables that are collinear (Pearson’s r of > 0.7) are delineated in red. Within collinear groups, one variable (identified with an asterisk) was selected prior to model fitting.

Supplementary Table 1

Woody plant cover statistics for African countries derived from data presented in Fig. 1. The average trend (slope of the linear regression between 1986 and 2016) of the area undergoing significant gains and losses in woody cover should be interpreted relative to the total percentage woody cover, average trend and unmasked area (Supplementary Fig. 1) for each country.

Country	Total			Gain		Loss	
	Woody cover (%)	Trend (% yr^{-1})	Unmasked area (km^2)	Area (km^2)	Trend (% yr^{-1})	Area (km^2)	Trend (% yr^{-1})
Angola	53	0.7	1023879	247131	0.8	28905	-0.6
Benin	42	0.6	63464	6929	0.6	374	-0.5
Botswana	28	0.8	618648	291435	0.8	6549	-0.3
Burkina Faso	19	0.4	105059	24446	0.4	2280	-0.4
Burundi	62	0.1	7100	546	0.5	405	-0.4
Cameroon	57	1.1	199047	27175	1.1	808	-0.7
Central African Republic	60	1.4	535743	221859	1.3	3600	-0.4
Chad	20	0.4	504490	59107	0.7	36507	-0.3
Congo	60	-0.3	71747	2359	0.6	5931	-0.7
Congo DRC	62	1.0	805462	195762	1.1	27197	-0.7
Djibouti	5	0.2	22019	3763	0.2	143	-0.2
Eritrea	11	0.0	63511	14375	0.2	5082	-0.5
Ethiopia	34	0.4	851864	199166	0.6	34544	-0.5
Gabon	73	0.2	19744	243	0.5	89	-0.5
Gambia	39	0.5	1115	197	0.6	8	-0.6
Ghana	44	0.1	112289	4714	0.9	4156	-0.8
Guinea	58	0.5	219488	20913	0.7	4539	-0.6
Guinea-Bissau	60	0.8	20890	2924	0.9	183	-0.6
Ivory Coast	61	0.5	155198	16654	0.8	4746	-0.7
Kenya	32	-0.1	477358	37970	0.4	32216	-0.7
Lesotho	37	0.5	34873	15330	0.5	287	-0.5
Liberia	77	0.8	35788	4699	0.8	20	-0.5
Madagascar	44	-0.6	492315	28858	0.5	76792	-1.0
Malawi	50	0.5	83995	25878	0.6	2730	-0.6
Mali	19	0.5	256609	48260	0.5	4149	-0.2
Mauritania	4	0.3	6760	2104	0.2	43	-0.2
Mozambique	60	0.3	747550	116220	0.7	53230	-0.6
Namibia	19	0.5	886784	254625	0.5	9884	-0.2
Niger	2	-0.1	207426	15690	0.2	36477	-0.2
Nigeria	44	0.8	279679	37073	0.9	3036	-0.5
Rwanda	65	0.0	3557	275	0.5	414	-0.4

Senegal	27	0.6	55462	8735	0.7	763	-0.4
Sierra Leone	69	0.3	49381	4563	0.7	2112	-0.6
Somalia	21	-0.1	544493	39083	0.4	79199	-0.4
South Africa	28	0.4	1214440	478366	0.5	31016	-0.4
South Sudan	43	1.2	518436	120047	1.1	3039	-0.6
Sudan	14	0.1	596673	53714	0.5	48360	-0.3
Swaziland	58	0.7	15672	5515	0.7	428	-0.5
Tanzania	55	0.6	700997	175749	0.8	32055	-0.6
Togo	47	0.6	27772	2407	0.8	476	-0.6
Uganda	57	1.1	63329	19481	1.1	764	-0.4
Zambia	57	0.5	676749	148146	0.7	26399	-0.6
Zimbabwe	46	0.7	399737	146043	0.7	4638	-0.6

99

Supplementary Table 2

Internal and external random forest (RF) regression validation accuracies for predicting fractional woody cover using time-series metrics derived from Landsat satellites. Internal accuracies are evaluated by the proportion of variance in the response variable explained (PVE) as well as the mean of square residuals (MSR) produced from cross-validation between in-bag and out-of-bag samples. External accuracies are evaluated by predicting against a testing dataset withheld during model construction. The adjusted R^2 of the linear regression between observed and predicted woody cover is presented.

	Internal		External
RF model	PVE	MSR	Adjusted R^2
Landsat 5 TM	0.935	0.152	0.915
Landsat 7 ETM+	0.917	0.161	0.924
Landsat 8 OLI	0.93	0.134	0.925

Supplementary references:

1 Gorelick, N. *et al.* Google Earth Engine: Planetary-scale geospatial analysis for everyone. *Remote Sens. Environ.* **202**, 18-27 (2017).

2 White, F. *Vegetation of Africa – A Descriptive Memoir to Accompany the UNESCO/AETFAT/UNSO Vegetation Map of Africa*. (Unesco, 1983).
