# Supplementary material for: Drivers of woody plant encroachment over Africa
Source: Nat Commun. 2018 Jun 11;9:2272. doi: 10.1038/s41467-018-04616-8 (PMC5995890; doi:10.1038/s41467-018-04616-8)
Supplement: Supplementary file 2 — Peer Review File [file 41467_2018_4616_MOESM2_ESM.pdf]

Editorial Note: This manuscript has been previously reviewed at another journal that is not operating a transparent peer review scheme. This document only contains reviewer comments and rebuttal letters for versions considered at *Nature Communications*.

REVIEWERS' COMMENTS:

Reviewer #1 (Remarks to the Author):

I have reread the manuscript several times, and have found it a useful piece of work and I recommend publication. The shear scale of the data makes it a very useful piece of work. It also reads much more smoothly and I am satisfied that the authors have tried to achieve a more balanced argument. The figures are very helpful and clear and provide much of the detail.
